# Health science students’ knowledge, attitude, and readiness toward health management learning in the Gulf Cooperation Council region: a multi-institutional study

**DOI:** 10.3389/fmed.2026.1720974

**Published:** 2026-02-13

**Authors:** Ali Artaman, Amjad Abu ElSamen, Hillani Tadesse Bekele, Heba Hijazi, Ghada Moh. Samir Elhessewi, Fatima Al-Ghadban, Mohaimen Mansur

**Affiliations:** 1 College of Natural and Health Sciences, Zayed University, Dubai, United Arab Emirates; 2 College of Medicine and Health Sciences, Khalifa University, Abu Dhabi, United Arab Emirates; 3 Department of Health Care Management, College of Health Sciences, University of Sharjah, Sharjah, United Arab Emirates; 4 Department of Health Management and Policy, Faculty of Medicine, Jordan University of Science and Technology, Irbid, Jordan; 5 College of Health and Rehabilitation Sciences, Princess Nourah bint Abdulrahman University, Riyadh, Saudi Arabia; 6 College of Public Health, Kuwait University, Safat, Kuwait; 7 Institute of Statistical Research and Training, University of Dhaka, Dhaka, Bangladesh

**Keywords:** curriculum development, Gulf Cooperation Council (GCC), health management education, leadership and decision-making, undergraduate students

## Abstract

**Background:**

Health management represents a core competency for healthcare professionals, especially in systems experiencing rapid reform and resource constraints. While traditionally overlooked in undergraduate curricula, it is vital for promoting efficiency, patient safety, and interprofessional collaboration.

**Methods:**

Four universities were partner sites for this classroom-based research: Zayed University (ZU), with two campuses, the University of Sharjah (US), Princess Noura Bint Abdul Rahman University (PNU), and Kuwait University. A total of 307 health science students were recruited from each university through email and in-class invitations from August 2022 to January 2024. Following the data check, score variables were created for general knowledge, attitude, and readiness. Univariate and multivariate logistic regression were performed to identify significant predictors of knowledge and attitude.

**Results:**

Students largely demonstrated a solid foundational understanding of core health management concepts. However, fewer students recognized the connection between health management and epidemiology. Those with prior exposure to health systems or economics courses were more likely to exhibit greater knowledge. Differences in knowledge levels were also observed across institutions. Positive attitudes toward pursuing a management role were more common among senior students but varied by academic background and university. Readiness to engage in health management learning was generally moderate and was higher among students with previous degree experience.

**Conclusion:**

Students generally acknowledged the value of health management and demonstrated moderate readiness to engage with the topic. Prior coursework and academic standing influenced both knowledge and attitude. Strengthening undergraduate curricula with foundational management content may enhance student preparedness for future roles.

## Background

Health management is a foundational pillar of public health that encompasses the administration of healthcare services, financial oversight, ethical governance, and human resource coordination (1). While often used interchangeably, health administration, health management, and health economics represent distinct yet interrelated domains. Health administration focuses on policy implementation and resource allocation (1), health management emphasizes operational performance and regulatory compliance (1), and health economics applies economic theory to address issues of resource efficiency and cost control (2).

Historically, health administration was formalized earlier than public health education at the undergraduate level, with programs first offered in the 1920s, while undergraduate public health degrees emerged in the 1970s (3). The Association of University Programs in Health Administration (AUPHA) outlines key competencies for health management education, including communication, professionalism, and systems knowledge (4). These competencies have evolved from self-reported assessments to include external evaluations, such as internship feedback and capstone assessments (4), strengthening the emphasis on measurable student outcomes.

Program structure and institutional alignment have also been shown to influence students’ graduate education trajectories. For instance, relocating a healthcare administration program from a college of business to a health sciences college significantly shifted students’ preferences toward healthcare-specific graduate programs like the MHA (5). Curriculum structure varies globally: South Korean programs emphasize theory and pathology, while UK curricula focus more on leadership, policy, and economics (6). This diversity necessitates greater attention to how program design affects student perception and readiness for management roles.

Despite an increased focus on competencies, gaps in knowledge and self-efficacy remain (7). Students nearing graduation often report lower confidence in domains like policy, strategy, and operations, despite relatively stronger perceptions of their skill-based competencies (7). Likewise, students’ career expectations may clash with program content; as freshmen often expect clinical training and are surprised by business-oriented coursework, while transfer students who have taken prerequisite courses report higher engagement and satisfaction (8). A commentary highlights a persistent ‘theory–policy–practice gap,’ noting that even medical students often lack adequate exposure to real-world issues of health policy and management, underscoring the need for curriculum integration that bridges these divides (9).

Pedagogical strategies significantly impact learning outcomes. Mixed-method instruction combining large lectures with small classes promotes collaboration and leadership skills, particularly in healthcare management training (10). Interactive technologies, such as Learning Catalytics, improve student engagement and self-reported learning in courses like health economics (11). Similarly, students participating in interprofessional education (IPE) experiences report improved confidence in communication and leadership (12). In line with this, curricular interventions that integrate interdisciplinary content, including Pharmacoeconomics and policy, have shown promising results in enhancing cross-professional competencies (13).

Across regions, however, management and health economics education remain fragmented. In South Africa and Botswana, qualitative studies reveal a lack of structured delivery and curricular integration, despite widespread acknowledgment of the importance of these subjects (14). Systematic reviews echo these findings, noting that while students value quality improvement and teamwork, attitudes toward cost containment and error management remain mixed and susceptible to the quality of educational interventions (15).

The need for evidence-based curriculum reform has been strongly articulated in recent literature. UK-based studies advocate for needs assessments and stakeholder-driven development of health economics competencies (16). Systematic reviews emphasize integrating cost-conscious care into healthcare training (17). Similarly, interprofessional education programs have demonstrated measurable benefits in strengthening management and teamwork competencies (18). Further, a systematic review of medical students’ attitudes toward medical leadership and management found that students held positive attitudes toward clinical practice guidelines, quality improvement techniques, and multidisciplinary teamwork (17). However, their attitudes were mixed regarding managed care, cost containment, and medical error. Additionally, educational interventions also affected the attitudes of the students. Medical students perceive a need for leadership and management education but identified a lack of curriculum time and disinterest in some activities as potential barriers to implementation (17).

Despite global acknowledgment of the importance of health management in health professional education, empirical research on undergraduate student readiness, especially in the Gulf Cooperation Council (GCC), remains scarce. Most existing studies are either discipline-specific or conducted in Western contexts. This study aims to address this gap by examining the knowledge, attitudes, and readiness of undergraduate health science students in the GCC toward health management education. By doing so, it offers an empirical foundation for strengthening curricula across interdisciplinary health programs and contributes to ongoing debates about how best to prepare the next generation of healthcare leaders. These insights can inform curriculum development to ensure that future health professionals are well-equipped with the competencies required for leadership and decision-making roles. This need aligns with findings from Hammad et al. (13), who emphasized the importance of expanding educational offerings in health economics and related fields across professional programs.

## Methods

### Study design

This study employed a cross-sectional exploratory design using a self-completed questionnaire to assess the knowledge, attitude, and readiness of undergraduate health science students for the Health Management course. Given the financial and time constraints, a convenience sampling strategy was adopted to recruit participants from Zayed University (Abu Dhabi and Dubai campuses) and Princess Noura Bint Abdul Rahman University (KSA), University of Sharjah, and Kuwait University.

### Sample size

Four universities were partner sites for this classroom-based research: Zayed University (ZU), with two campuses, the University of Sharjah (US) and Princess Noura Bint Abdul Rahman University (PNU), and Kuwait University. A total of 307 health science students were recruited from each university through email and in-class invitations. The students were invited to respond to a self-administered online questionnaire in the first week of classes. The data was collected through Google Forms, which was fully anonymous. The inclusion criterion is being an undergraduate health science student, while the exclusion criteria were as follows: being a graduate student and an undergraduate student of a program other than health science.

### Data collection instrument

The questionnaire was developed after reviewing relevant literature (13). Content and face validity of the questionnaire was assessed by experts. Furthermore, pretest followed by a pilot test of the questionnaire was conducted. The online questionnaire consisted of 43 closed-ended questions, divided into four sections: demographics, knowledge toward health management learning, attitude toward health management learning, and readiness for health management learning. The demographics section included six questions assessing the year of study, the start year of their degree program, whether it is the student’s first university degree program, the university of enrollment, and the number of registered credits. The full questionnaire has been included in Supplementary File 1.

### Knowledge and attitude toward health management learning

Students’ knowledge of health management learning was measured using nine questions, which were then added up to generate a knowledge score variable. These questions were mainly related to the terminologies used in health management, such as efficiency and effectiveness, and the knowledge required for the practice of health administration and health management. Students were classified as having sufficient knowledge if the number of correctly answered questions was above the average of all students; otherwise, they were not. The attitude variable, on the other hand, was determined based on whether students intended to pursue health management as a full-time or part-time career. Those who want to pursue health management career were considered to have a positive attitude.

### Readiness to learn health management

Students’ readiness to learn health management was measured based on their prior coursework, such as taking courses in public health or health systems and a policy or business course (economics, finance or management), and a technical course (statistics or computer science). The students were considered ready if they have taken at least three of the courses. With a total score out of 7, if students scored 3 or more, they are considered ready to learn health management, otherwise not.

### Ethical clearance

This study was conducted according to the guidelines of the Declaration of Zayed University and approved by the Research Ethics Committee (REC), Office of Research at Zayed University with an approval code of ZU22_102_F. Ethical concerns of this study were taken care of according to the Declaration of Helsinki (2001). All participants joined the study voluntarily after signing an informed consent form. The form described the goals and objectives of the study and clearly explained that the survey is strictly anonymous and confidential, and the participants were informed that they could withdraw from completing the survey at any point, without giving a reason, and without consequences of any kind.

### Data analysis

The collected data were cleaned using IBM SPSS Statistics version 29, and analysis was conducted using R Studio. Descriptive statistics were used to summarize categorical variables as percentages. The Chi-square test was conducted to assess the relationship between student characteristics and both knowledge of health management and attitude toward health management learning. Multivariate logistic regression analysis was conducted, adjusting for key background variables (year of study, university, first-degree program or not, completion of more than 15 credit hours). Additionally, other independent/explanatory variables with a *p*-value of 0.15 or below on the bivariate Chi-square tests were included in the model to reduce the risk of excluding potentially important predictors, consistent with recommendations for exploratory regression modeling. Variables with a *p*-value less than 0.05 were considered to be statistically significant.

## Results

### Respondents’ characteristics

As illustrated in Table 1, a total of 307 students participated in our study. Most students were from Zayed University (53%), followed by Princess Noura University (38%). The majority of the students were fourth (37%) and third year (35%) university students. 90% of the students reported starting school between 2019 and 2021, while 78% of them reported that it was their first degree. Additionally, 45% were taking more than 15 credit hours during the study, while 13% of the students reported taking less than 10 credit hours.

**Table 1:** Background characteristics of the respondents.

Characteristics	***N* = 307***^1^*
Starting year	
2017	5 (1.6%)
2018	13 (4.2%)
2019	97 (32%)
2020	79 (26%)
2021	97 (32%)
2022	16 (5.2%)
Year of study	
First	3 (1.0%)
Second	78 (25%)
Third	107 (35%)
Fourth	113 (37%)
Fifth	6 (2.0%)
University	
ZU Abu Dhabi	26 (8.5%)
ZU Dubai	135 (44%)
PNU	118 (38%)
Sharjah	21 (6.8%)
Kuwait	7 (2.3%)
Degree program	
First degree program	240 (78%)
Number of credits currently taking	
Taking >15 credits	137 (45%)
Taking < 10credits	39 (13%)
Prior courses taken	
Have taken prior ‘public health’ course	285 (93%)
Have taken prior ‘health system’ course	131 (43%)
Have taken prior ‘policy’ course	65 (21%)
Have taken prior ‘management’ course	93 (30%)
Have taken prior ‘economics’ course	39 (13%)
Have taken prior ‘finance’ course	33 (11%)
Have taken prior ‘statistics’ course	237 (77%)
Have taken prior ‘computer science’ course	150 (49%)

^1^*n* (%).

### Knowledge about health management

As presented in Table 2, students’ knowledge of health management was assessed using nine questions that measured their basic understanding of common terminologies used in health management and the foundational concepts necessary for the practice of health administration. The knowledge items were intentionally designed to assess foundational conceptual literacy rather than applied managerial competence. This approach aligns with the exploratory objective of evaluating baseline readiness for health management learning among undergraduate students, rather than mastery of advanced competencies. Among all students, 90% reported knowing the meaning of the term ‘efficiency,’ while 96% reported knowing the meaning of the term effectiveness, and 97% reported knowing the meaning of the word ‘strategy.’ Moreover, 74% of them agreed that ‘Quality’ is a topic of health management, while only 49% agreed that Epidemiology is a toolbox for health management. In terms of the practice of health administration. Regarding the practical aspects of health administration, 62% of the students reported the necessity of mathematics knowledge, while 68% of them emphasized the importance of having informatics knowledge. Furthermore, 81% of students indicated that a hospital manager must be familiar with ‘strategic’ management.

**Table 2:** Knowledge of Health Management descriptive statistics.

Characteristic	*N* = 307*^1^*
I know the meaning of the word, ‘efficiency’	
No	31 (10%)
Yes	276 (90%)
I know the meaning of the word, ‘effectiveness’	
No	11 (3.6%)
Yes	296 (96%)
I know the meaning of the word, ‘strategy’	
No	9 (2.9%)
Yes	298 (97%)
Quality is a topic of health management	
No	6 (2.0%)
Yes	226 (74%)
I do not know	75 (24%)
Epidemiology is a toolbox for health management	
No	10 (3.3%)
Yes	150 (49%)
I do not know	147 (48%)
The practice of ‘health administration’ require some knowledge of ‘mathematics’	
No	30 (9.8%)
Yes	189 (62%)
I do not know	88 (29%)
The practice of ‘health administration’ require some knowledge of ‘informatics’	
No	20 (6.5%)
Yes	209 (68%)
I do not know	78 (25%)
A hospital manager needs to know ‘strategic’ management	
No	2 (0.7%)
Yes	248 (81%)
I do not know	57 (19%)
Is Population health the same as public health?	
No	113 (37%)
Yes	126 (41%)
I do not know	68 (22%)

^1^*n* (%).

Students were classified as having sufficient knowledge if the number of correctly answered questions was above the average score of all respondents. The average knowledge score across all respondents was 6.57 out of 9. Overall, 57% of the participants scored above average. To assess the relationship between knowledge about health management and student characteristics, a Chi-square test was conducted, as shown in Supplementary Table 1. The results showed that several variables were significantly associated with knowledge, including university (*p* = 0.011), taking prior health system courses (*p* < 0.001), and prior economics courses (*p* = 0.021). Approximately 70% of students who took a prior health systems course had sufficient general knowledge of epidemiology, compared to fewer than 50% of those who had not taken such a course. Similarly, there was a 20%-point difference in the proportion of students with sufficient knowledge between those who had taken an economics course and those who had not.

The overall model fit was assessed using the Hosmer-Lemeshow goodness-of-fit test. The results indicated acceptable model fit for both logistic regression models (*p* > 0.05). After adjusting for other variables, as shown in Table 3, the odds of correctly answering a sufficient number of knowledge questions were 2.5 times higher among students who had taken a prior health systems course compared to those who had not (AOR: 2.52, *p*-value: 0.001). Students who considered epidemiology as the backbone of public health had 3.5 times higher odds of demonstrating better knowledge of health management (OR: 3.54, *p*-value: 0.002). Similarly, students who considered health policy as the backbone of public health had 2.4 times higher odds of better knowledge (OR: 2.39, *p*-value: 0.032). It’s noted that some institutional estimates showed wide confidence intervals, reflecting limited subgroup sample sizes and should therefore be interpreted cautiously.

**Table 3:** Multivariate logistic regression between knowledge of health management and respondents’ characteristics.

Characteristic	OR^1^	95% CI^1^	*p*-value
Year of study			
First and second	—	—	
Third	0.65	0.33, 1.27	0.2
Fourth and fifth	1.22	0.50, 3.01	0.7
University			
ZU Abu Dhabi	—	—	
ZU Dubai	0.88	0.34, 2.20	0.8
PNU	0.96	0.33, 2.75	>0.9
Sharjah	3.68	0.79, 21.0	0.11
Kuwait	0.14	0.01, 1.10	0.10
First degree			
No	—	—	
Yes	0.91	0.49, 1.68	0.8
Taking >15 credits			
No	—	—	
Yes	0.94	0.55, 1.61	0.8
Have taken prior health system course			
No	—	—	
Yes	2.52	1.45, 4.47	**0.001**
Have taken prior policy course			
No	—	—	
Yes	0.95	0.45, 2.01	0.9
Have taken prior economics course			
No	—	—	
Yes	1.63	0.64, 4.36	0.3
Have taken prior management course			
No	—	—	
Yes	1.43	0.76, 2.71	0.3
Which one do you consider as a backbone of public health			
Healthcare economics	—	—	
Health policy	2.39	1.09, 5.41	**0.032**
Epidemiology	3.54	1.60, 8.11	**0.002**
Want calculation in health management course			
No	—	—	
Yes	1.48	0.88, 2.48	0.14

^1^OR = Odds Ratio, CI = Confidence Interval.

Bold values indicate statistically significant results (*p* < 0.05).

### Attitude toward health management learning

Attitude toward health management was operationalized as career orientation (interest in full-time or part-time management roles), rather than affective or cognitive attitude dimensions. As shown in Table 4, the majority of the participants (98%) reported that health management is useful for their future careers, and 99% agreed that health management is important for public health students. As for their career preferences, only 47% of students expressed interest in becoming a full-time manager after graduation, while 52% of them preferred to pursue part-time management consultant roles. Moreover, 47% of the students considered epidemiology as the backbone of public health, while 37% of them considered health policy to be the backbone of public health. On the other hand, the majority of the students (82%) believed that COVID-19 had made health management a popular field.

**Table 4:** Attitude toward health management education.

Characteristic	*N* = 307*^1^*
Can health management be useful for your future career?	
No	7 (2.3%)
Yes	300 (98%)
I do not know	0 (0%)
Is health management important for public health student?	
No	3 (1.0%)
Yes	304 (99%)
Do you want to be a full-time ‘manager’ after graduation from your current program?	
No	162 (53%)
Yes	145 (47%)
I do not know	0 (0%)
Do you want to be a part-time ‘management consultant’ after graduation from your current program?	
No	146 (48%)
Yes	161 (52%)
I do not know	0 (0%)
Which one do you consider as the ‘backbone’ of ‘public health’?	
Health policy	115 (37%)
Healthcare economics	48 (16%)
Epidemiology	144 (47%)
Did COVID-19 make ‘health management’ a ‘popular’ field?	
No	5 (1.6%)
Yes	253 (82%)
I do not know	49 (16%)
Do you think one health management course is enough for a Bachelor of Public Health or nutrition student?	
No	148 (48%)
Yes	159 (52%)
I do not know	0 (0%)
Which mode of (health management) teaching do you prefer?	
Online	101 (33%)
On-campus	206 (67%)
Which type of course assessment do you prefer?	
Test	211 (69%)
Essay	96 (31%)
Do you want ‘guest speaker’ for the health management class?	
No	99 (32%)
Yes	208 (68%)
Do you want calculations for introductory health management course?	
No	180 (59%)
Yes	127 (41%)
I do not know	0 (0%)
Do you want Web portal (a website with several links related to health administration) for learning introductory health management?	
No	84 (27%)
Yes	223 (73%)
Is management like marketing	
No	157 (51%)
Yes	26 (8.5%)
May be	124 (40%)

^1^*n* (%).

Regarding academic preferences, 52% of the students felt that one health management course was enough for a Bachelor of Public Health or nutrition student. The preferred mode of delivery for health management courses was on-campus instruction (67%), while 69% of the students preferred to have test assessments rather than essays. 68% of the students wanted guest speakers during their classes, and 59% of the students did not want a calculation within the health management course. Additionally, 73% of the students wanted a web portal with several links related to health administration for learning introductory health management, and 51% disagreed with the idea that management is similar to marketing.

The attitude variable was categorized into ‘positive’ and ‘not positive’ based on whether the respondents expressed interest in a full-time or part-time health management career. Overall, 73% of the respondents indicated a preference for a full-time or part-time role in health management. A chi-square test was conducted to assess the association between student characteristics and attitude toward health management course, as shown in Supplementary Table 2. The results found that university (*p* = 0.011) and their answer regarding the backbone of public health (*p* = 0.002) were significantly associated with their attitudes toward health management. Knowledge about health management was only marginally associated with attitude toward health management, at 10% level of significance (*p* = 0.069).

After adjusting for other variables using logistic regression, as shown in Table 5, respondents from PNU had around 80% lower odds of having a positive attitude toward health management (AOR: 0.21, *p*-value: 0.21) compared to students from the reference university, ZU Abu Dhabi. Additionally, fourth- and fifth-year students were three times more likely to have a positive attitude toward health management (AOR: 3.05, *p*-value: 0.036) compared to students in their first and second years. Students who had taken a previous economics course had 70% lower odds of having a positive attitude toward health management (AOR: 0.31, *p*-value: 0.015) in comparison to those who had not taken such a course. General knowledge of health management was associated with higher odds of having a positive attitude toward the subject, although not statistically significant at the highest levels (AOR: 2.55, *p*-value: 0.041).

**Table 5:** Attitude (positivity) logistic regression.

Characteristic	OR^1^	95% CI^1^	*p*-value
Year of study			
First and second	—	—	
Third	1.31	0.61, 2.80	0.5
Fourth and fifth	3.05	1.10, 8.94	0.036
University			
ZU Abu Dhabi	—	—	
ZU Dubai	0.71	0.21, 2.02	0.5
PNU	0.21	0.06, 0.70	**0.015**
Sharjah	8.08	0.86, 194	0.10
Kuwait	0.71	0.09, 6.89	0.7
First degree program			
No	—	—	
Yes	0.63	0.31, 1.23	0.2
Taking > 15 credits			
No	—	—	
Yes	0.78	0.42, 1.43	0.4
Have taken prior public health course			
No	—	—	
Yes	0.74	0.14, 3.06	0.7
Have taken prior economics course			
No	—	—	
Yes	0.31	0.12, 0.79	**0.015**
Which one do you consider as a backbone of public health			
Healthcare economics	—	—	
Health policy	2.01	0.88, 4.61	0.10
Epidemiology	2.27	1.00, 5.16	0.049
Correct knowledge questions			
Below 5	—	—	
5–6	2.55	1.03, 6.31	**0.041**
Above 6	1.72	0.74, 3.91	0.2

^1^OR = Odds Ratio, CI = Confidence Interval.

Bold values indicate statistically significant results (*p* < 0.05).

### Readiness to learn health management

Readiness for health management learning was defined based on whether the respondents had taken a prior health course (public health or health system), a policy course/ a business course (economics, finance or management), and a technical course (statistics or computer science). Overall, 68% of the participants were considered ready to learn health management. A Chi-square test found that readiness was significantly associated with having their degree program as first-degree program or not as shown in Supplementary Table 3.

## Discussion

Given the exploratory nature of this multi-institutional study, the findings should be interpreted as indicative patterns intended to inform curriculum development rather than definitive assessments of competence. This study examined health science students’ knowledge, attitude, and readiness for health management courses in the GCC region. Our findings revealed that almost half of the respondents (57%) had an above-average knowledge score in health management. This was in line with the findings of a study with 190 students at Pennsylvania State University, where students reported positive self-assessments of their knowledge in health administration (7). Taking a prior health system course was associated with higher odds of answering the knowledge questions correctly, emphasizing the importance of including courses related to health management within the curriculum. This finding was consistent with a study among 2,648 health professions students in Jordan, which found that students who had taken health economics or Pharmacoeconomics course were more likely to report higher median knowledge score (13). However, we found no significant association between the year of study and knowledge score, differing from the results from a study at Pennsylvania State University where most senior students reported having little or no knowledge in health law, marketing, operations, strategy, accounting, policy and finance (13). The variance could be attributed to the difference in data collection tool and due to the small number of sample size among the Pennsylvania State University study. A Malaysian study among medical students also found that year of study and knowledge of health policy were significantly associated with their involvement in health policy roles (19).

The observed negative association between prior economics coursework and positive attitudes toward health management may reflect a misalignment between the abstract or quantitative framing of economics courses and students’ expectations of applied, healthcare-oriented leadership roles. This finding should be interpreted cautiously and does not imply that economics education is detrimental to health management learning. Rather, it highlights the importance of aligning course framing and pedagogy with students’ expectations of applied, healthcare-specific management competencies. Additionally, our respondents had a positive attitude toward health management learning, with nearly all participants agreeing that it is useful for their future careers (98%) and that health management is important for public health students (99%). These results are consistent with a qualitative study in four medical schools in Southern Africa, where participants acknowledged the importance of teaching management intentionally and explicitly in medical schools (14). Similarly, a Jordanian study found that those who had taken prior Health economics or Pharmacoeconomics courses recognized the importance of health economics in patient care. Pharmacy students showed the highest interest in health economics, followed by medical, dental and nursing students (13). Additionally, a survey conducted at a public university among medical students in Malaysia found that most medical students were willing to learn health policy (81%) and agreed that teaching health policy (84%) should be compulsory (19). As for their career preferences, wanted to be either a full-time manager or a part-time management consultant after graduation. This finding is aligned with a qualitative study in Iran, where 26 medical students reported that one of their primary motivations to enter a Master of Public Health program was preparing for the management of health systems (20).

Half of our participants believed that one health management course was enough for a Bachelor of Public Health or nutrition degree. The mode of study was one of the key predictors of satisfaction in public health courses (21). More than half of the respondents preferred learning health management on campus and favored test assessments over essays. Moreover, 68% of the respondents wanted guest speakers during their classes and 59% of the students do not want calculations within health management course. And 73% of the students wanted a web portal with several links related to health administration for learning introductory health management. We found that students’ attitude toward health management learning was weakly associated with the knowledge score of health management. Senior students (fourth- and fifth-year students) had three times the odds of having a positive attitude toward health management compared to younger students. Surprisingly, students who had taken previous economics course had lower odds of having positive attitude toward health management.

The top five competencies in health care management included professionalism, understanding the healthcare environment, business knowledge, communication, business skills (5). It is essential to incorporate these competencies when designing a health management curriculum for undergraduate students. A Malaysian study suggested including health policy courses earlier in the first or second year of medical school (19). Depending on the prior coursework (health course, policy course and business course, and technical course), 68% of students were considered ready to learn health management. The only factor that was associated with readiness was whether the health management program was not their first-degree program. Additionally, our study found no significant association between readiness for learning health management and students’ knowledge or attitudes toward health management. Taken together, these findings provide preliminary, exploratory evidence to inform curriculum development and highlight areas where more rigorous, theory-driven and longitudinal research is needed to guide the integration of health management education in undergraduate health programs.

### Limitations

This study presents several limitations that should be considered when interpreting its results. Firstly, due to its cross-sectional design, it is not possible to establish causality, and the findings are limited to associations observed at a single time point. Secondly, the use of convenience sampling and the disproportionate representation of certain institutions affect the sample’s representativeness, thereby reducing the generalizability of the results to all undergraduate health science programs within the GCC region. As a result, comparisons between institutions should be viewed with caution.

Thirdly, reliance on self-reported responses may have introduced recall bias or social desirability bias, especially concerning measures related to knowledge and attitudes. Moreover, the knowledge assessment was intended to evaluate basic conceptual understanding rather than practical management skills, while the operationalization of attitude as career orientation fails to encompass broader psychological constructs such as self-efficacy or perceived difficulty.

Lastly, small sample sizes in some university subgroups led to wide confidence intervals and less precise regression estimates. Overall, these limitations frame the current study as an exploratory, multi-institutional needs assessment rather than a conclusive evaluation of competency or preparedness. Future studies should adopt representative sampling methods, employ validated multidimensional tools, and utilize longitudinal or intervention-based designs to more effectively assess the progression of health management knowledge, attitudes, and preparedness over time.

## Conclusion

This study found that a considerable proportion of health science students demonstrated above-average knowledge of health management, particularly among those who had completed prior coursework in health systems. While students’ attitudes toward health management were moderately associated with knowledge, they were more strongly influenced by academic seniority, suggesting that increased exposure to healthcare learning environments may foster more favorable views. Interestingly, students’ readiness to engage in health management education appeared to be shaped less by their knowledge or attitudes and more by their broader academic experience, such as having completed a prior degree.

These findings highlight the importance of integrating health systems and management content early within undergraduate curricula. Doing so may strengthen students’ foundational understanding and enhance their engagement with health leadership topics. Future research should focus on validating the current findings through the use of standardized instruments and larger, more diverse samples across the GCC region. Such efforts will provide more robust, generalizable insights to guide the development of responsive and effective educational strategies in health management.

## Data Availability

The raw data supporting the conclusions of this article will be made available by the authors, without undue reservation.
