# Supplementary material for: Health science students’ knowledge, attitude, and readiness toward health management learning in the Gulf Cooperation Council region: a multi-institutional study
Source: Front Med (Lausanne). 2026 Feb 13;13:1720974. doi: 10.3389/fmed.2026.1720974 (PMC12947382; doi:10.3389/fmed.2026.1720974)
Supplement: Supplementary file 1 [file Supplementary_file_1.pdf]

Week 1

Public Health 2 class survey

* Required

1. Are you a 'public health' or 'nutrition' student? *

Mark only one oval.

- ( ) yes
- ( ) No

2. What year of the bachelor (undergraduate) program are you in? *

Mark only one oval.

- ( ) 1
- ( ) 2
- ( ) 3
- ( ) 4
- ( ) 5

3. Which year did you start your current undergraduate education? *

Mark only one oval.

- 2017
- 2018
- 2019
- 2020
- 2021
- 2022

4. Is this your 'first' university degree program? *

Mark only one oval.

- Yes
- No

5. Prior to this semester, did you ever take a 'public health' course? *

Mark only one oval.

- Yes
- No

6. Prior to this semester, did you ever take a 'statistics' course? *

Mark only one oval.

- Yes
- No

7. Prior to this semester, did you ever take a course 'not' titled 'statistics' but included a 'considerable' number of lectures on statistical concepts (probability, hypothesis testing, etc)? *

Mark only one oval.

- Yes
- No

8. Prior to this semester, did you ever take a 'management' or 'business' course? *

Mark only one oval.

- Yes
- No

9. Prior to this semester, did you ever take a course 'not' titled 'management' but included a 'considerable' number of lectures on management/ administration? *

Mark only one oval.

- Yes
- No

10. Prior to this semester, did you ever take an 'economics' course? *

Mark only one oval.

- Yes
- No

11. Prior to this semester, did you ever take a 'policy' course? *

Mark only one oval.

- Yes
- No

12. Prior to this semester, did you ever take an 'accounting' or a 'finance' course? *

Mark only one oval.

- Yes
- No

13. Prior to this semester, did you ever take a 'health system' course? *

Mark only one oval.

- Yes
- No

14. Prior to this semester, did you ever take a 'global health' or 'international health' course? *

Mark only one oval.

- Yes
- No

15. Prior to this semester, did you ever take a 'computer science' or 'information technology' course? *

Mark only one oval.

- Yes
- No

16. Do you have more than 15 credits of courses this semester? *

Mark only one oval.

- Yes
- No

17. Do you have less than 10 credits of courses this semester? *

Mark only one oval.

- Yes
- No

18. Do you know the meaning of the word, 'efficiency'? *

Mark only one oval.

- Yes
- No

19. Do you know the meaning of the word, 'effectiveness'? *

Mark only one oval.

- Yes
- No

20. Do you know the meaning of the word, 'strategy'? *

Mark only one oval.

- Yes
- No

21. Is 'quality' a topic of 'health management'? *

Mark only one oval.

- Yes
- No
- I do not know

22. Is 'health management' important for 'public health' student? *

Mark only one oval.

- Yes
- No

23. Did COVID-19 make 'health management' a 'popular' field? *

Mark only one oval.

- Yes
- No
- I do not know

24. Does epidemiology provide a toolbox for 'health management'? *

Mark only one oval.

- Yes
- No
- I do not know

25. Does the practice of 'health administration' require some knowledge of 'mathematics'? *

Mark only one oval.

- Yes
- No
- I do not know

26. Does the practice of 'health administration' require some knowledge of 'statistics'? *

Mark only one oval.

- Yes
- No
- I do not know

27. Does the practice of 'health administration' require some knowledge of informatics (information technology, computation, computer science)? *

Mark only one oval.

- Yes
- No
- I do not know

28. Do you think one (1) health management course (Public Health 2) is enough for a bachelor of public health or nutrition student? *

Mark only one oval.

- Yes
- No

29. For the introductory health management course, do you want number crunching (number calculations)? *

Mark only one oval.

- Yes
- No

30. Can health management be 'useful' for your future career? *

Mark only one oval.

- Yes
- No

31. Do you want to be a full-time 'manager' after graduation from your current program? *

Mark only one oval.

- Yes
- No

32. Do you want to be a part-time 'management consultant' after graduation from your current program? *

Mark only one oval.

- Yes
- No

33. Do you know a healthcare executive (manager, director)? *

Mark only one oval.

- Yes
- No

34. Which one do you consider as the 'backbone' of 'public health'? *

Mark only one oval.

- Health policy
- Healthcare economics
- Epidemiology

35. Which mode of (health management) teaching do you prefer? *

Mark only one oval.

- Online
- On-campus

36. Which type of course assessment do you prefer? *

Mark only one oval.

- Test
- Essay

37. Do you want 'guest speaker' for the health management class? *

Mark only one oval.

- Yes
- No

38. Do you want Web portal (a Website with several links related to health administration) for learning Public Health 2? *

Mark only one oval.

- Yes
- No

39. Does a 'public health professional' need to know 'strategic' management? *

Mark only one oval.

- Yes
- No
- I do not know

40. Does a 'hospital manager' need to know 'strategic' management? *

Mark only one oval.

- Yes
- No
- I do not know

41. Is 'population health' the same as 'public health'? *

Mark only one oval.

- Yes
- No
- I do not know

42. Is management like 'marketing'? *

Mark only one oval.

- Yes
- No
- Maybe

This content is neither created nor endorsed by Google.

Google Forms
