# Supplementary material for: Health science students’ knowledge, attitude, and readiness toward health management learning in the Gulf Cooperation Council region: a multi-institutional study
Source: Front Med (Lausanne). 2026 Feb 13;13:1720974. doi: 10.3389/fmed.2026.1720974 (PMC12947382; doi:10.3389/fmed.2026.1720974)
Supplement: Supplementary file 2 [file Supplementary_file_2.docx]

Supplementary Table 1: Bivariate relationship between knowledge about health management and respondents’ characteristics.

| **Characteristic** | **Sufficient**, N = 176*^1^* | **Not Sufficient**, N = 131*^1^* | **p-value***^2^* |
| --- | --- | --- | --- |
| Year of study |  |  | 0.4 |
| First & second | 43 (53%) | 38 (47%) |  |
| Third | 59 (55%) | 48 (45%) |  |
| Fourth & fifth | 74 (62%) | 45 (38%) |  |
| University |  |  | **0.011** |
| ZU Abu Dhabi | 15 (58%) | 11 (42%) |  |
| ZU Dubai | 74 (55%) | 61 (45%) |  |
| PNU | 68 (58%) | 50 (42%) |  |
| Sharjah | 18 (86%) | 3 (14%) |  |
| Kuwait | 1 (14%) | 6 (86%) |  |
| First degree program |  |  | 0.9 |
| No | 39 (58%) | 28 (42%) |  |
| Yes | 137 (57%) | 103 (43%) |  |
| Taking >15 credits |  |  | >0.9 |
| No | 98 (58%) | 72 (42%) |  |
| Yes | 78 (57%) | 59 (43%) |  |
| Have taken prior public health course |  |  | 0.13 |
| No | 16 (73%) | 6 (27%) |  |
| Yes | 160 (56%) | 125 (44%) |  |
| Have taken prior health system course |  |  | **<0.001** |
| No | 85 (48%) | 91 (52%) |  |
| Yes | 91 (69%) | 40 (31%) |  |
| Have taken prior policy course |  |  | 0.11 |
| No | 133 (55%) | 109 (45%) |  |
| Yes | 43 (66%) | 22 (34%) |  |
| Have taken prior management course |  |  | 0.054 |
| No | 115 (54%) | 99 (46%) |  |
| Yes | 61 (66%) | 32 (34%) |  |
| Have taken prior economics course |  |  | **0.021** |
| No | 147 (55%) | 121 (45%) |  |
| Yes | 29 (74%) | 10 (26%) |  |
| Have taken prior accounting or finance course |  |  | 0.058 |
| No | 152 (55%) | 122 (45%) |  |
| Yes | 24 (73%) | 9 (27%) |  |
| Have taken prior statistics course |  |  | 0.6 |
| No | 38 (54%) | 32 (46%) |  |
| Yes | 138 (58%) | 99 (42%) |  |
| Have taken prior computer science course |  |  | 0.4 |
| No | 86 (55%) | 71 (45%) |  |
| Yes | 90 (60%) | 60 (40%) |  |
| Health management is important for public health students |  |  | 0.077 |
| No | 0 (0%) | 3 (100%) |  |
| Yes | 176 (58%) | 128 (42%) |  |
| Health management is useful for future career |  |  | 0.5 |
| No | 3 (43%) | 4 (57%) |  |
| Yes | 173 (58%) | 127 (42%) |  |
| Which one do you consider as a backbone of public health |  |  | **0.041** |
| Health policy | 66 (57%) | 49 (43%) |  |
| Healthcare economics | 20 (42%) | 28 (58%) |  |
| Epidemiology | 90 (63%) | 54 (38%) |  |
| Want calculation in health management course |  |  | 0.055 |
| No | 95 (53%) | 85 (47%) |  |
| Yes | 81 (64%) | 46 (36%) |  |
| *^1^*n (%) | | | |
| *^2^*Pearson's Chi-squared test; Fisher's exact test | | | |

Supplementary Table 2: A bivariate relationship between attitude (positivity) towards health management and respondent characteristics.

| **Characteristic** | **Yes**, N = 224*^1^* | **No**, N = 83*^1^* | **p-value***^2^* |
| --- | --- | --- | --- |
| Year of study |  |  | 0.9 |
| First & second | 59 (73%) | 22 (27%) |  |
| Third | 80 (75%) | 27 (25%) |  |
| Fourth & fifth | 85 (71%) | 34 (29%) |  |
| University |  |  | 0.011 |
| ZU Abu Dhabi | 21 (81%) | 5 (19%) |  |
| ZU Dubai | 103 (76%) | 32 (24%) |  |
| PNU | 75 (64%) | 43 (36%) |  |
| Sharjah | 20 (95%) | 1 (4.8%) |  |
| Kuwait | 5 (71%) | 2 (29%) |  |
| First degree |  |  | 0.7 |
| No | 50 (75%) | 17 (25%) |  |
| Yes | 174 (73%) | 66 (28%) |  |
| Taking >15 credits |  |  | 0.6 |
| No | 126 (74%) | 44 (26%) |  |
| Yes | 98 (72%) | 39 (28%) |  |
| Have taken prior public health course |  |  | 0.14 |
| No | 19 (86%) | 3 (14%) |  |
| Yes | 205 (72%) | 80 (28%) |  |
| Have taken prior health system course |  |  | 0.7 |
| No | 127 (72%) | 49 (28%) |  |
| Yes | 97 (74%) | 34 (26%) |  |
| Have taken prior policy course |  |  | 0.3 |
| No | 180 (74%) | 62 (26%) |  |
| Yes | 44 (68%) | 21 (32%) |  |
| Have taken prior management course |  |  | 0.5 |
| No | 154 (72%) | 60 (28%) |  |
| Yes | 70 (75%) | 23 (25%) |  |
| Have taken prior economics course |  |  | 0.086 |
| No | 200 (75%) | 68 (25%) |  |
| Yes | 24 (62%) | 15 (38%) |  |
| Have taken prior accounting or finance course |  |  | >0.9 |
| No | 200 (73%) | 74 (27%) |  |
| Yes | 24 (73%) | 9 (27%) |  |
| Have taken prior statistics course |  |  | 0.4 |
| No | 54 (77%) | 16 (23%) |  |
| Yes | 170 (72%) | 67 (28%) |  |
| Have taken prior computer science course |  |  | 0.2 |
| No | 110 (70%) | 47 (30%) |  |
| Yes | 114 (76%) | 36 (24%) |  |
| Which one do you consider as a backbone of public health |  |  | 0.002 |
| Health policy | 87 (76%) | 28 (24%) |  |
| Healthcare economics | 25 (52%) | 23 (48%) |  |
| Epidemiology | 112 (78%) | 32 (22%) |  |
| Correct knowledge questions |  |  | 0.069 |
| below 5 | 20 (57%) | 15 (43%) |  |
| 5-6 | 74 (77%) | 22 (23%) |  |
| above 6 | 130 (74%) | 46 (26%) |  |
| *^1^*n (%) | | | |
| *^2^*Pearson's Chi-squared test; Fisher's exact test | | | |

Supplementary Table 3: A bivariate relationship between readiness, knowledge, and attitude towards health management learning.

| **Characteristic** | **Ready**, N = 127*^1^* | **Not**, N = 180*^1^* | **p-value***^2^* |
| --- | --- | --- | --- |
| Year of study |  |  | 0.063 |
| First & second | 25 (31%) | 56 (69%) |  |
| Third | 51 (48%) | 56 (52%) |  |
| Fourth & fifth | 51 (43%) | 68 (57%) |  |
| UNIVERSITY |  |  | 0.15 |
| ZU Abu Dhabi | 10 (38%) | 16 (62%) |  |
| ZU Dubai | 57 (42%) | 78 (58%) |  |
| PNU | 44 (37%) | 74 (63%) |  |
| Sharjah | 14 (67%) | 7 (33%) |  |
| Kuwait | 2 (29%) | 5 (71%) |  |
| First degree program |  |  | 0.009 |
| No | 37 (55%) | 30 (45%) |  |
| Yes | 90 (38%) | 150 (63%) |  |
| Taking >15 credits |  |  | 0.5 |
| No | 73 (43%) | 97 (57%) |  |
| Yes | 54 (39%) | 83 (61%) |  |
| Correct knowledge questions |  |  | 0.6 |
| below 5 | 15 (43%) | 20 (57%) |  |
| 5-6 | 36 (38%) | 60 (63%) |  |
| above 6 | 76 (43%) | 100 (57%) |  |
| Positive attitude |  |  | 0.5 |
| Yes | 95 (42%) | 129 (58%) |  |
| No | 32 (39%) | 51 (61%) |  |
| *^1^*n (%) | | | |
| *^2^*Pearson's Chi-squared test; Fisher's exact test | | | |
